# Cancer-Associated Fibroblasts: Understanding Their Heterogeneity

**DOI:** 10.3390/cancers12113108

**Published:** 2020-10-24

**Authors:** Kévin Louault, Rong-Rong Li, Yves A. DeClerck

**Affiliations:** 1 Division of Hematology, Oncology and Blood and Marrow Transplantation, Children’s Hospital Los Angeles, Los Angeles, CA 90027, USA; 2 Department of Pediatrics, Keck School of Medicine of the University of Southern California, Los Angeles, CA 90027, USA; 3 The Saban Research Institute, Children’s Hospital Los Angeles, Los Angeles, CA 90027, USA; 4 Department of Pharmacology and Pharmaceutical Sciences, School of Pharmacy, University of Southern California, Los Angeles, CA 90033, USA; lirongro@usc.edu; 5 Department of Biochemistry and Molecular Biology, Keck School of Medicine of the University of Southern California, Los Angeles, CA 90033, USA

**Keywords:** cancer-associated fibroblasts (CAFs), tumor microenvironment (TME), heterogeneity, mesenchymal stromal cells (MSCs), cancer, biomarkers

## Abstract

**Simple Summary:**

Cancer-associated fibroblasts (CAFs) play an important contributory role in the microenvironment of tumors. They originate from different cells, have multiple pro-and anti-tumorigenic functions in tumors and their presence is variable among cancer types. Recently, there has been evidence that CAFs represent a highly heterogeneous group of cells that can now be characterized and identified at the single cell level. This review article summarizes our recent understanding of the highly heterogeneous nature of the origin, phenotype and function of CAFs and how such understanding will lead to a more precise approach to target or use CAFs and their precursor cells in the treatment of cancer.

**Abstract:**

The tumor microenvironment (TME) plays a critical role in tumor progression. Among its multiple components are cancer-associated fibroblasts (CAFs) that are the main suppliers of extracellular matrix molecules and important contributors to inflammation. As a source of growth factors, cytokines, chemokines and other regulatory molecules, they participate in cancer progression, metastasis, angiogenesis, immune cell reprogramming and therapeutic resistance. Nevertheless, their role is not fully understood, and is sometimes controversial due to their heterogeneity. CAFs are heterogeneous in their origin, phenotype, function and presence within tumors. As a result, strategies to target CAFs in cancer therapy have been hampered by the difficulties in better defining the various populations of CAFs and by the lack of clear recognition of their specific function in cancer progression. This review discusses how a greater understanding of the heterogeneous nature of CAFs could lead to better approaches aimed at their use or at their targeting in the treatment of cancer.

## 1. Introduction

Over the last few decades, scientific evidence in support of Paget’s "Seed and Soil" theory has been obtained through an improved understanding of the cellular and molecular hallmarks of cancer cells (the Seed) and an in-depth characterization of the highly complex tumor microenvironment (TME; the Soil) [1,2,3]. Four major components of the TME [4] have been identified: 1) an immune component known as the tumor immune microenvironment (TIME), composed of a large variety of innate immune cells such as tumor-associated macrophages (TAMs), natural killer (NK) cells, neutrophils, mast cells, dendritic cells (DCs), myeloid-derived suppressive cells (MDSCs) and of adaptative immune cells including CD4+ T helper lymphocytes (Th), CD8+ cytotoxic T cells, NK-T cells, γδT cells, regulatory T cells (Treg), and B cells [5,6,7,8]; 2) a vascular component consisting of micro-vascular and lymphatic endothelial cells (ECs) and pericytes [9,10]; 3) an extracellular matrix (ECM) component made of diverse collagen molecules, glycoproteins and proteoglycans [11,12]; and 4) a less well defined stromal component that includes non-immune cells of mesenchymal origin such as mesenchymal stromal cells (MSCs) and cancer-associated fibroblasts (CAFs), which are the focus of this review [13,14]. 

Fibroblasts are characterized by their contractile and ECM remodeling activities [15]. These cells become activated and proliferate during inflammation, wound repair and fibrosis, and during malignant progression where they were first reported to accelerate the growth of epithelial tumors by L.W. Chung et al. and were later designated as cancer-associated fibroblasts (CAFs) [14,16,17,18]. For some time, CAFs were considered a homogeneous population of ECM-producing stromal cells, responsible for the stiff 3-D architecture of a tumor. However, multiple studies over the last two decades have indicated that CAFs are not innocent bystanders in tumors and can have pro- or anti-tumorigenic functions [19,20,21]. These opposite activities raise the question of whether CAFs represent a homogeneous and well-defined population of stromal cells or rather are members of a heterogeneous group of stromal cells that differ in their origin, phenotype, function and presence in different types of cancers. Recent evidence indicates that it is the latter rather than the former situation. In this review, we discuss how these differences could explain the various and sometimes contradictory roles of CAFs in cancer progression, and how such heterogeneity makes their consideration in cancer therapy challenging.

## 2. Origins of CAF Heterogeneity 

**Figure 1:**
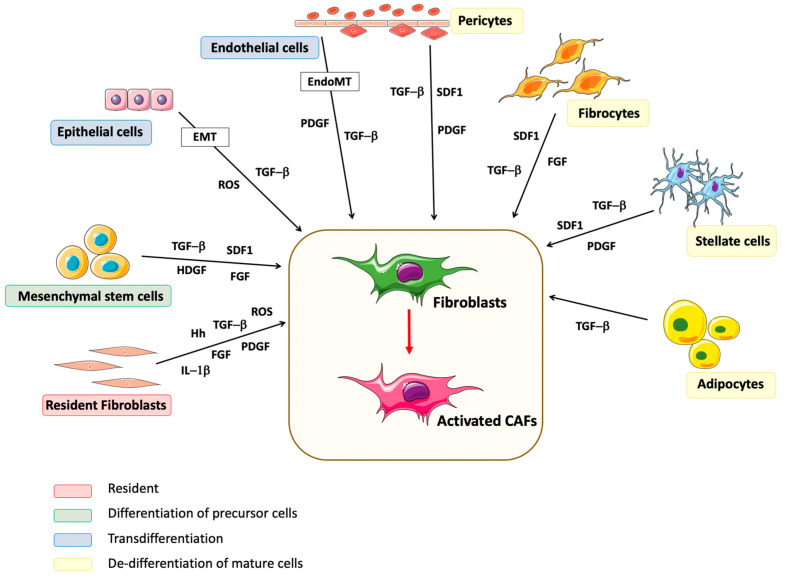
Origin of Cancer-Associated Fibroblasts. The different cellular sources for CAFs are shown, colors indicate the type of origin. Factors involved in the recruitment, differentiation and activation of these cells into CAFs are indicated for each cell type.

There is now strong experimental evidence that CAFs can originate from a variety of cells such as resident fibroblasts, a variety of precursor cells that differentiate, mature cells that de-differentiate or transdifferentiate, or even tumor cells (Figure 1) [22]. 

Resident fibroblasts are located in the tissue of origin of the primary tumor and are the main source of CAFs in a tumor. Transforming growth factor beta 1 (TGF-β1), secreted by stromal and tumor cells, is the main factor promoting the mobilization of resident fibroblasts and their activation into CAFs [23,24]. TGF-β1, through SMAD-dependent and independent pathways, activates fibroblasts into CAFs, expressing alpha-smooth muscle actin (α-SMA, a.k.a. ACTA2), periostin (POSTN), α-fibroblast activation protein (αFAP, a.k.a. dipeptidylpeptidase IV) and fibroblast specific protein-1 (FSP-1, a.k.a. S100A4), and produces type I collagen [25]. Other factors secreted by tumor cells such as platelet-derived growth factor (PDGF), fibroblast growth factor (FGF), sonic hedgehog (SHH) and interleukin (IL) 1-β promote the conversion of resident fibroblasts into CAFs through activation of signaling pathways such as the extracellular signal regulated kinase (ERK), Shh/Smo (smoothened), or nuclear factor kappa-B (NFκB) pathways [26,27,28,29]. CAFs are characterized not only by their expression of α-SMA, αFAP and FSP-1, but also by their production of vascular endothelial growth factor (VEGF) and cytokines like IL-6 and IL-8 [26,27]. Hypoxia is another factor that promotes the activation of resident fibroblasts into CAFs through the accumulation of reactive oxygen species (ROS) and activation of the hypoxia inducible factor (HIF)-1α-mediated signaling pathway [30,31,32]. In pancreatic cancer, vitamin A and D are responsible for reprogramming CAFs from a quiescent state into an active pro-tumorigenic state via activation of SMAD signaling or the renin-angiotensin system, increasing the proliferation and metabolism of CAFs [33,34,35] Moreover, activated fibroblasts secrete TGF-β1 and the C-X-C chemokine ligand-12 (CXCL-12; a.k.a. stromal-derived factor (SDF) -1), which both act through autocrine and paracrine mechanisms to initiate and maintain their myofibroblasts phenotype and their tumor-promoting function [36].

CAFs also originate from a variety of precursor cells recruited by tumor cells at the sites of primary growth or metastasis. Among these, MSCs are an important source of CAFs and can provide up to 20% of the CAF population in a tumor [37]. They express similar markers as the CAFs derived from resident fibroblasts, i.e., α-SMA, vimentin (VIM) and αFAP, along with MSC markers including CD90 (a.k.a. THY1), CD105 (endoglin) and CD73 (a.k.a. 5’-nucleotidase) [37,38]. The recruitment of MSCs and their activation into CAFs are stimulated by CXCL-12 and TGF-β secreted by tumor cells [39,40,41]. Furthermore, hepatoma-derived growth factor (HDGF) and IL-1β promote the secretion by MSCs of pro-tumorigenic cytokines that stimulate tumor progression [37,42,43]. 

CAFs can also derive from mature cells. Through TGF-β-mediated epithelial to mesenchymal transition (EMT), epithelial cells differentiate into functional CAFs expressing FSP-1 and αFAP [44,45]. ECs contribute to the pool of CAFs through a similar process known as EndoMT, driven by TGF-β and SMAD signaling [46]. Pericytes, adipocytes, fibrocytes and stellate cells are all sources of CAFs. They are recruited in tumors by TGF-β and CXCL-12, and activated into CAFs by TGF-β or PDGF via mechanisms similar to those activating resident fibroblasts [46,47,48,49,50,51,52]. It has also been demonstrated that cancer cells, in particular cancer stem cells, can be a source of CAFs under the action of TGF-β [22,53].

## 3. Phenotypic and Functional Heterogeneity

**Figure 2:**
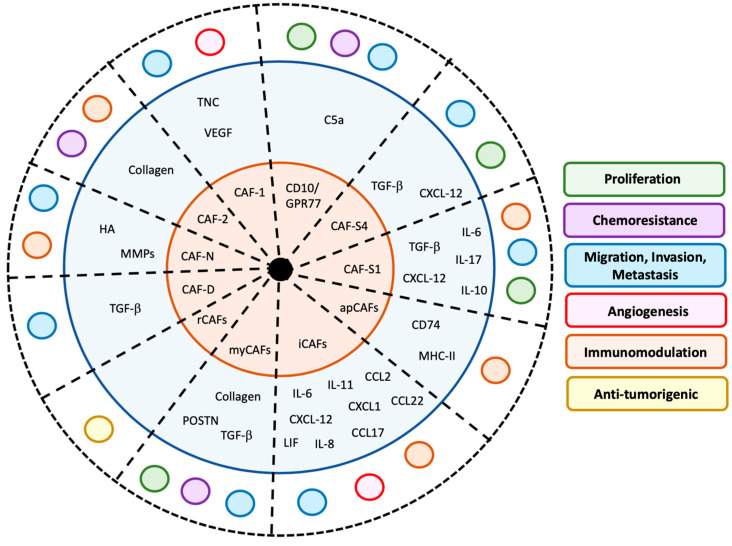
Heterogenous Function and Phenotype of Cancer-Associated Fibroblasts. The inner circle lists subtypes of CAFs reported by various groups of investigators. The middle circle indicates markers and proteins secreted by each subtype and in the outer circle, the functions attributed for each subtype are indicated by the colored circles representing one among the six CAF functions listed in the margin.

CAFs represent a heterogeneous population of cells. Major challenges in defining the sub-populations of CAFs have been the lack of consistency in identifying specific robust markers and difficulties in defining the association between subgroups and their specific function in cancer. However, a more comprehensive picture emerges based on a review of the most recent literature (Figure 2 and Table 1). 

### 3.1. Phenotypic Markers Associated with Specific CAFs Activities

α-SMA has long been the main marker used to characterize CAFs [18], despite being expressed by most mesenchymal cells including normal fibroblasts, myo-fibroblasts, myocytes and pericytes [74,75]. CAFs were later defined by the absence of expression of epithelial markers (cytokeratin, E-cadherin), ECs marker (CD31) and myeloid marker (CD45), and by the expression of mesenchymal markers such as VIM, FSP-1, PDGF receptor beta (PDGFRβ) and αFAP [18,76,77]. Although highly expressed in CAFs, these proteins are also expressed by mesodermal cells (αFAP), myeloid cells (FSP-1), pericytes (PDGFRβ) and normal fibroblasts (FSP-1, PDGFRβ) [78,79], reflecting their lack of specificity. 

However, more recent studies in a variety of murine cancer models and human tumors have now identified and characterized subpopulations of CAFs with specific functions based on surface markers, secreted proteins and transcriptome. R. Kalluri‘s laboratory was the first to bring attention to the heterogeneity among CAFs by describing two sub-populations of murine pro-tumorigenic CAFs in breast and pancreatic cancer models [54]. The first sub-population, labeled CAF-1, consists of CAF-expressing FSP-1, that promotes metastatic colonization by tumor cells [54,55,56]. In an orthotopic mouse model of breast cancer, they show that the production of tenascin by these FSP-1+ CAFs promotes VEGF-A-mediated angiogenesis and metastasis [55]. The second sub-population, called CAF-2, is characterized by the expression of α-SMA, neural/glial antigen 2 (NG2) and PDGFRβ [60]. This subtype of CAFs is a source of type I collagen, contributing to the formation of a fibrotic connective tissue barrier that prevents tumor infiltration by cytotoxic T lymphocytes [57]. Two different sub-populations of CAFs, designated CAF-N (normal) and CAF-D (divergent) were later described in human oral squamous cell carcinoma (OSCC). CAF-N secrete hyaluronic acid (HA) and matrix metalloproteinases (MMPs), promoting tissue invasion by cancer cells and by fibroblasts, and creating a HA-rich and immunosuppressive ECM, while CAF-D are a source of TGF-β that induces EMT in cancer cells and promotes cell migration [58,59]. In human colorectal tumors, two other sub-populations of CAFs were reported based on their transcriptome, CAF-A, rich in MMP2, αFAP and COL1A2 (type 1 collagen α−chain 2) mRNA, and CAF-B, rich in α-SMA, PDGF-A and TAGLN (transgelin) mRNA, but their function was not determined [60].

In human pancreatic ductal adenocarcinoma (PDAC) tumors and in transgenic mice engineered to carry KRas and p53 mutations under the control of the pancreatic specific Cre promoter (KPC), different subpopulations of CAFs were identified based on their expression of meflin, a glycosylphosphatidylinositol-anchored protein expressed by MSCs that maintains their undifferentiated state. Meflin-poor CAFs (pCAFs) have a pro-tumorigenic function, whereas meflin-rich CAFs (rCAFs) suppress PDAC progression by acting on ECM remodeling, hypoxia and cancer initiation [61,62]. The concept that some subpopulations of CAFs exert their function through the production of ECM proteins whereas other subpopulations of CAFs act as inflammatory cells is suggested by some recent reports. In mice xenotransplanted with PDAC tumors, pancreatic stellate cells (PSC) were shown to differentiate into α-SMA+ myofibroblasts, designated myCAFs, producing collagen and TGF-β, or into inflammatory CAFs having an immunomodulatory function, designated iCAFs secreting IL-6, IL-11 and leukemia inhibitory factor (LIF) that promote immune escape [63]. Using single cell RNA-sequencing analysis (scRNA-seq), a new powerful tool to further define CAF heterogeneity, it has been shown that iCAFs are characterized by the production of inflammatory cytokines and chemokines such as *IL-6, IL-8, CXCL1, CXCL12* and other proteins like complement factor D (*CFD*), lamin A/C (*LMNA*), podoplanin (*PDPN*), and dermatopontin (*DPT*), whereas myCAFs are characterized by the expression of *ACTA2, PDPN, TAGLN*, tropomyosin 1 and 2 (*TPM1, TPM2*) and *POSTN* [64,65,66]. iCAFs promote metastasis, angiogenesis and have an immunomodulatory function through their production of cytokines and chemokines. iCAFs secrete CCL17 and CCL22 that recruit Treg cells in tumors, resulting in an immunosuppressive microenvironment [80], and CCL2 and CXCL12 that recruit monocytes and promote their maturation into MDSCs that inhibit CD8+ T lymphocytes [81,82]. In contrast, myCAFs through the production of ECM proteins and TGF-β induce EMT and promote tumor cell proliferation, invasion and metastasis. A specific subtype of myCAFs expressing leucine-rich repeat containing 15 (LRRC15) is associated with chemoresistance to therapy targeting programmed death ligand-1 (PD-L1) in patients [66]. Another subtype of CAFs with an important immunomodulatory function has recently been reported in PDAC. This subpopulation of CAFs, designated antigen presenting-CAFs (apCAFs), expresses PDPN and COL1A1 as iCAFs and myCAFs but also major histocompatibility complex (MHC) class II family of proteins such as histocompatibility 2, class II antigen A alpha and beta 1 (H2-Aa, H2-Ab1), and CD74 that present antigen epitopes to their receptor on CD4+/CD8+ T lymphocytes. This binding inhibits activation and proliferation of lymphocytes, with apCAFs acting as a decoy [64,67].

In tumor samples from human breast adenocarcinoma, Costa et al. identified four subtypes of CAFs, designated CAF-S1 to CAF-S4, based on the expression of six markers, including integrin β1 (ITGB1, a.k.a. CD29), αFAP, PDGFRβ, FSP-1, α-SMA and caveolin 1 (Cav1). They defined CAF-S1 as strongly positive for all six markers, CAF-S2 as negative for all markers, CAF-S3 as rich in ITGB1, FSP-1 and PDGFRβ, and CAF-S4 as expressing α-SMA in addition to the three markers present in CAF-S3 [68]. Whereas the tumorigenic functions of CAF-S2 and S3 have not been defined, CAF-S1 and CAF-S4 secrete TGF-β and CXCL12 that activate NOTCH signaling in tumor cells, promoting proliferation and invasion [69]. CAF-S1 also have an immunosuppressive activity through the secretion of IL-6, IL-17, IL-10 and CXCL12 that induce the inhibition of CD4+ T lymphocytes, as well as the activation and the proliferation of Treg lymphocytes [68,70]. A recent transcriptomic analysis by scRNA-seq from the same group indicates a further level of heterogeneity among CAF-S1, also suggestive of the highly diverse spectrum of CAF functions, as it identified eight new clusters, each expressing specific genes coding ECM proteins (cluster 0, ecm-myCAFs), detoxification pathway (cluster 1, detox-iCAFs), IL signaling (cluster 2, IL-iCAFs), TGFβ signaling pathway (cluster 3, TGFβ-myCAFs), wound healing (cluster 4, wound-myCAFs), IFNγ (cluster 5, IFNγ-iCAFs), IFNαβ (cluster 6, IFNαβ-myCAFs), and acto-myosin pathway (cluster 7, acto-myCAFs). Interestingly, CAFs from clusters 0 and 3, characterized by extracellular matrix proteins and TGFβ signaling respectively, are indicative of primary resistance to immunotherapies and act synergistically with ecm-myCAF upregulating PD-1 and CTLA4 protein levels in regulatory T lymphocytes (Tregs), which, in turn, increases TGFβ-myCAF cellular content. These observations in breast cancer were also validated in head, neck and lung cancers [71]. Moreover, Kieffer et al. have also shown correlations between the different clusters and PDAC subtypes. Clusters 0, 3, 4, 6 and 7 are associated with myCAFs, especially ecm-myCAFs with LRRC15-myCAFs; whereas clusters 1, 2 and 5 are associated with iCAFs, especially IFNγ-iCAFs with apCAFs. The subgrouping of CAF-S1 in different clusters can also explain the diversity of the CAF activities described earlier.

In patient-derived xenograft (PDX) tumors of breast adenocarcinoma, other investigators have described subpopulations of CAFs expressing the G protein-coupled C5a receptor 77 (GPR77, a.k.a. C5AR2) and membrane metallo-endopeptidase (MME, a.k.a. CD10) that promote tumor formation and chemoresistance by providing a survival niche for cancer stem cells. CD10+GPR77+ CAFs are driven by persistent NF-κB activation, which is maintained by complement signaling via GPR77. Furthermore, CD10+GPR77+ CAFs promote successful engraftment of PDXs, and targeting these CAFs with a neutralizing anti-GPR77 antibody abolishes tumor formation and restores tumor chemosensitivity via the production of IL-6 and IL-8 [72]. ScRNA-seq analysis in murine models of breast cancer and human samples also suggest that different subpopulations of CAFs are spatially separated. Following a negative selection strategy combined with scRNA-seq of mesenchymal cells from a genetically engineered mouse model of breast cancer, Bartoschek et al. [73] define three distinct subpopulations of CAFs: vascular (vCAFs), matrix (mCAFs) and development (dCAFs). vCAFs express EC markers and are located in the peri-vascular niche, whereas mCAFs express matricellular proteins and are descendants of resident fibroblasts and dCAFs express stem cells genes (Scgr1, Sox9 and Sox10) shared with the tumor epithelium. Gene profiles for each CAF subtype correlate to distinctive functional programs. 

The summary above highlights the significant progress made in our understanding of the heterogeneity of the CAF population and in our ability to recognize specific subpopulations defined by their expression of specific surface markers, production and secretion of proteins and transcriptomic profiles. Whether there is an overlap among the different subpopulations reported by several laboratories in pancreatic cancer or breast cancer is uncertain and needs to be further investigated. Nevertheless, it is clear that these subpopulations of CAFs have different functions in cancer, which are far-reaching, extending from direct effect on tumor cell proliferation, survival, chemoresistance, invasion, and metastasis to effects mediated by changes in the ECM, the vasculature and in immune cells in the TME. Table 1 represents an attempt to link CAF subtypes with these specific functions based on the review of the recent literature. 

### 3.2. Mechanisms of CAF Functions

In this section, we briefly summarize our current understanding of the mechanisms by which CAFs exert each of the six main recognized functions as depicted in Figure 2 (Table 2). 

#### 3.2.1. Effects of CAFs on Tumor Proliferation

CAFs secrete multiple factors such as TGF-β1, CXCL-12, FGF, POSTN, osteopontin (OPN), hepatocyte growth factor (HGF), IL-6 and IL-22 that directly stimulate the proliferation of tumor cells in vitro and tumor growth in vivo via activation of their respective signaling pathways, i.e., integrin/FAK-src (POSTN), Wnt/β-catenin (HGF and OPN), PI3K/mTOR (CXCL-12, HGF and IL-22), MAPK (IL-6, TGF-β and FGF) or Hippo (EVs) [83,84,85,86,87,88,89,90]. These factors also increase the survival of tumor cells via upregulation of anti-apoptotic proteins like B-cell lymphoma-2 (BCL-2) and myeloid cell leukemia-1 (MCL-1) or downregulation of pro-apoptotic proteins like BCL-2 Associated X protein (BAX) [83,84]. 

#### 3.2.2. Chemoresistance

Several studies have indicated that CAFs play an important role in chemoresistance via different mechanisms such as secretion of cytokine and miRNAs, increase in cancer stem cells (CSCs), and decreased drug bioavailability via production of a stromal barrier [134]. In breast, colorectal and pancreatic tumors, CAFs increase the resistance of cells to chemotherapeutic agents like gemcitabine and doxorubicin and to combination chemotherapy like doxorubicin, 5-fluorouracil and cisplatin through the production of IL-6, IL-17A, PDGF and insulin like growth factor (IGF) that activate the NF-κB and ERK pathways in tumor cells, promoting the stabilization of anti-apoptotic proteins and the proliferation of cancer stem cells [91,92,93,94,95]. For example, IL-6 increases the survival of breast cancer cells via ERK signaling that stabilizes MCL-1 [38,91]. CAFs are also a source of extracellular vesicles (EVs) that contain regulatory microRNAs (miR) like CmiR-98-5p and miR-522. When EVs are captured by tumor cells, miRNAs can promote resistance to cisplatin by downregulating cyclin-dependent kinase inhibitor (CDKN)1A and ferroptosis, a newly described programmed cell death mechanism characterized by the accumulation of lipid peroxides and a lack of cellular anti-oxidant response [100,101]. In PDAC and breast cancer, the presence of a physical barrier, induced by secretion of hyaluronan and collagen by CAFs, reduces drug delivery. In particular, hyaluronan creates a high interstitial fluid pressure, limiting drug diffusion and causing vascular collapse [96,97,98,99]. 

#### 3.2.3. Migration, pro-Invasive and Metastatic Activity of CAFs 

CAFs directly stimulate tumor cell migration and invasion through the secretion of TGF-β, IL-32, PDGF and FGF that induce EMT, actin polymerization/depolymerization and cell motility [102,103,104,105]. In a model of breast cancer, IL-32 secreted by CAFs promotes metastasis in vivo via activation of β-integrin-mediated p38MAPK signaling in tumor cells, inducing loss of cell-cell adhesion and promoting cell migration [104]. In a similar model, the complement 3a (C3a) protein secreted by CAFs induces an autocrine loop that stimulates PI3K/Akt signaling and the secretion of TGF-β, HGF and PDGF that promote EMT, invasion and lung metastasis in tumor cells [103]. In an orthotopic murine model of hepatocellular carcinoma, the Hippo-Yes-associated protein (YAP) signaling pathway induced by CAFs in cancer cells increases EMT and stimulates actin cytoskeleton polymerization via the upregulation of Rho-GTPase activating proteins (ARHGAP29), both effects resulting in promotion of invasion and metastasis in cancer cells [106].

#### 3.2.4. Activity of CAFs on Angiogenesis

CAFs secrete multiple angiogenic molecules like VEGF, PDGF, CXCL-12 or HGF that promote ECM remodeling, the proliferation of ECs and the recruitment of ECs and pericytes to the tumor [107,108,109]. PDGF and VEGF have also an autocrine effect on CAFs, further stimulating the production of additional pro-angiogenic factors such as IL-6, IL-8 and placental growth factor (PGF) [110,111]. In a murine hepatocellular carcinoma xenograft model, TGF-β and CXCL-12 secreted by CAFs induce the production of proteins such as VE-cadherin, MMP-2 and laminin-5γ2 that promote vascular mimicry [112,113]. 

#### 3.2.5. Immunosuppressive Activity of CAFs 

CAFs affect the recruitment, polarization and function of immune cells in the TIME [135,136]. They participate in the recruitment of several immune cells, in particular monocytes and myeloid cells, via the production of chemokines such as IL-1β, CCL22, CXCL-12 and CCL2. For example, CXCL1, CXCL5 and IL-8 secreted by FGF-activated CAFs induce the recruitment of macrophages to tumors [80,114,115]. CAFs can also affect the polarization of immune cells (TAMs, T cells and neutrophils) [116,117,118,119]. When co-cultured with CAFs, TAMs polarize from anti-tumorigenic M1 to pro-tumorigenic M2 via the production of IL-10 by CAFs or from M2 to M1 via IL-12, thereby regulating the inflammatory response [120]. TGF-β, CXCL1 and IL-10 secreted by CAFs inhibit the function of NK cells, CD8+ T lymphocytes, Th1 lymphocytes and DCs [121,137,138]. These cytokines upregulate the expression of transcription factors such as T-bet and NOX4 in tumor cells, contributing to immune escape [118,121,122,123,124]. CAFs also suppress immune cells through direct contact by expressing βig-h3, a TGFβ-induced RGD-containing surface protein that binds to the integrin β3 (CD61) present on CD8+ T lymphocytes, inhibiting their cytotoxic function [125]. 

#### 3.2.6. Anti-Tumorigenic Activities of CAFs

Contrary to their pro-tumorigenic functions, CAFs can also have a negative impact on tumor progression. In mouse models of PDAC, the population of CAFs expressing α-SMA exhibit increased activation of Shh signaling that inhibits the production of VEGF, CXCL-12 and IL-8, which affects tumor growth, angiogenesis and immunosuppression [126,127]. In sarcoma and prostatic adenocarcinoma, CAFs secrete TGF-β, IL-6 and tumor necrosis factor-alpha (TNF-α) that activate YAP1/TAZ and NFκB signaling in tumor cells, resulting in inhibition of tumor cell proliferation [128,129,130,131]. In a murine model of OSCC, a sub-population of CAFs, characterized by low α-SMA expression and secretion of bone morphogenetic protein 4 (BMP4), was reported to decrease cell proliferation and inhibit cancer stem cells [132]. In intestinal tumors, overactivation of NFκB signaling in CAFs inhibits tumor growth and angiogenesis in vivo, and the depletion of this CAF sub-population induces the increased production of HGF, IL-6 and FGF and increased the recruitment and activity of Treg cells [133].

## 4. Heterogeneous Presence of CAFs in Human Cancers

**Figure 3:**
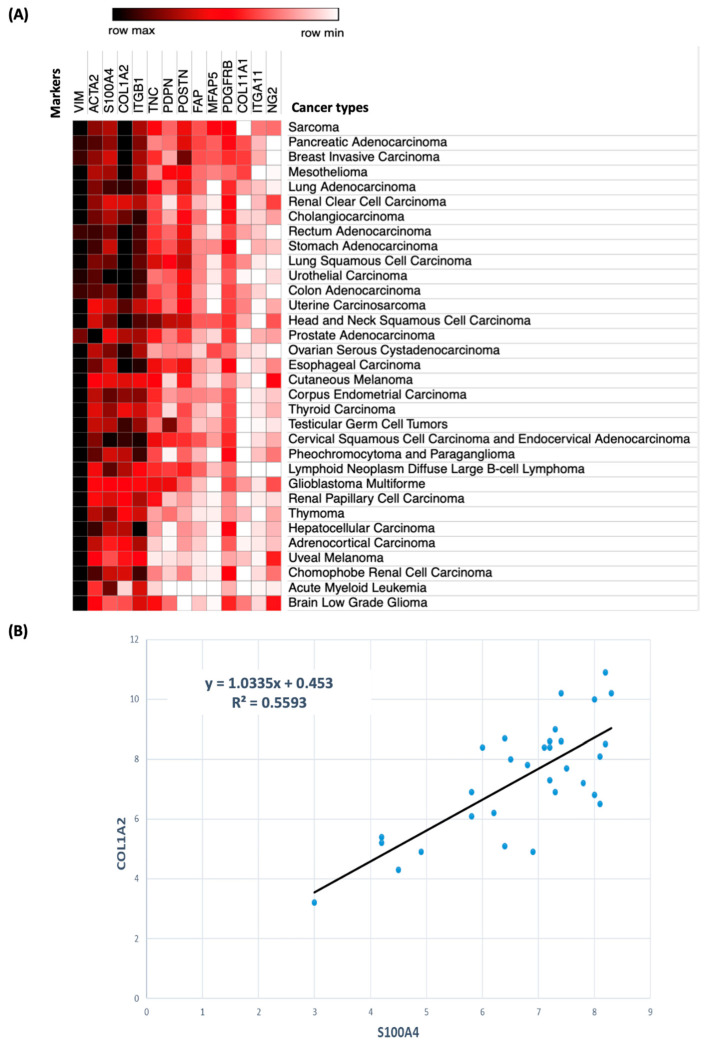
In silico gene expression analysis of 14 genes expressed by CAFs in human tumors. (**A**). Heat map representation of the expression of 14 genes (column) among 33 types of cancer (row). Data were generated by Gene Expression Profiling Interactive Analysis (GEPIA) of The Cancer Genome Atlas (TCGA) database; (**B**). Correlation analysis between the expression of S100A4 and COL1A2 in cancer.

Although CAFs have been reported to be present in most solid tumors, there is a significant degree of variability regarding their abundance among cancers, with pancreatic adenocarcinoma, breast carcinoma, lung adenocarcinoma and kidney renal clear cell carcinoma, being typically heavily infiltrated with CAFs, and leukemia, lymphoma and brain tumors being mostly devoid of CAFs. An in silico analysis of transcriptomics data obtained from the Gene Expression Profiling Interactive Analysis (GEPIA) databases (http://gepia.cancer-pku.cn) [139] for the mRNA expression of several CAF markers (*VIM, ACTA2, S100A4, COL1A2, ITGB1, TNC, PDPN, POSTN, FAP, MFAP5* (microfibril associated protein 5, a.k.a. *MAGP2*), *PDGFRβ, COL11A1* (collagen type 11 alpha chain 1), *ITGA11* (integrin alpha 11), and *NG2*) performed by our group illustrates this point (Figure 3A). Vimentin was highly expressed in all types of cancers, including in acute myeloid leukemia, suggesting that it is not a specific marker of CAFs. In contrast, four transcripts, *ACTA2, S100A4, COL1A2* and *ITGB1*, had the highest level of expression in cancers known to have a high proportion of CAFs, whereas low mRNA levels of these four genes were found in uveal melanoma, acute myeloid leukemia and low-grade brain glioma [79,140]. These data are consistent with other studies demonstrating that S100A4 and α-SMA (a.k.a. *ACTA2*) are overexpressed in most pancreatic adenocarcinoma and breast cancers [141,142]. We also found a positive correlation (r^2^ = 0.55) between the mRNA expression of *S100A4*, a gene that reflects the inflammatory function of CAFs [143] and *COL1A2*, a gene reflecting their effect on the stiffness of the tumor ECM [144], suggesting that these two functions of CAFs are closely associated (Figure 3B).

This analysis also points to other markers for tumor-specific functions (Figure 3A). *POSTN* is expressed more abundantly in invasive breast carcinoma, lung adenocarcinoma and squamous cell carcinoma, colon adenocarcinoma and head and neck squamous cell carcinoma (HNSCC). *POSTN* encodes for periostin, which was highly expressed in these cancers as demonstrated by immunohistohemistry (IHC) [145]. In contrast, CAF-poor tumors such as low-grade glioma and acute myeloid leukemia had low *POSTN* expression. Periostin was initially identified in osteoblasts, but is also secreted by CAFs. Present in the ECM, it promotes tumor cell adhesion, proliferation and migration through integrin binding and activation of the YAP signaling pathway, and through the secretion of cytokines such as IL-6 and TGF-β that promote immune escape and EMT [89,146,147]. Podoplanin (PDPN) is a mucin-type transmembrane glycoprotein and a lymphatic vessel marker. We found *PDPN* to be abundantly expressed in mesothelioma, HNSCC, lung squamous cell carcinoma and testicular germ cell tumors. This observation is consistent with an analysis of 662 tumors, examined by IHC for PDPN expression, which revealed that PDPN is expressed by stromal α−SMA+, VIM**^-^** myofibroblasts [148]. PDPN expression by CAFs may represent a predictive marker of lymphatic/vascular spread [148]. In breast cancer, high presence of PDPN+ CAFs is positively correlated with tumor size, invasive potential and poor prognosis in patients [149,150]. PDPN+ CAFs also have an unfavorable prognostic value in lung squamous cell carcinoma [151,152]. *MFAP5* was strongly expressed in fibroblastic cancers such as breast, ovarian, sarcoma, pancreatic and lung cancers. MFAP5 is a 25-kD microfibril-associated glycoprotein present in the ECM that interacts with integrins such as α_V_β_3_ expressed by ECs and with other ECM molecules such as collagen IV. MFAP5 promotes ECs motility and the rearrangement of their cytoskeleton via Notch signaling [153,154]. Recent studies show a correlation between *MFAP5* and *ACTA2* expression in CAFs, suggesting that they may identify a new subtype of CAFs [79,155]. In bladder cancer and oral squamous cell carcinoma, MFAP5 secreted by CAFs activates NOTCH2/HEY1, ERK and PI3K signaling pathways directly, promoting the proliferation, migration and invasion of cancer cells [155,156]. In murine xenografted models of ovarian cancer, MFAP5 secreted by CAFs upregulates lipoma-preferred partner (LPP) in ECs via FAK/ERK signaling, which promotes paclitaxel chemoresistance and angiogenesis [157]. In bladder and breast cancers, high expression of MFAP5 in CAFs correlated with high-grade malignancy, presence of metastasis and unfavorable clinical outcome [155]. In murine models of ovarian and PDAC cancers, inhibition of MFAP5 with monoclonal antibodies is associated with a decrease in the number of CAFs and microvessels, but also with increased sensitivity to paclitaxel [158]. 

Although this in silico transcriptomic analysis is informative, it does not take into consideration the potentially significant heterogeneity in CAF distribution within a single tumor where different sub-populations of CAFs with pro-and anti-tumorigenic function could co-exist. This aspect is well illustrated in the recent report by Bartocheck et al. discussed above [73], and by Öhlund et al. showing that myCAFs are located in close proximity to tumor cells, while iCAFs are distant [63]. A deeper understanding of the spatial heterogeneity of CAF in cancer will be obtained with further studies at the single cell level. 

## 5. Challenges in Targeting CAF Activity

Evidence supporting a pro-tumorigenic role for CAFs summarized in this review suggests that targeting CAFs in human cancer could be a valuable, albeit challenging strategy. With the increased ability to further characterize subtypes of CAFs associated with specific functions, we can anticipate a more ‘precise’ approach to targeting CAFs in cancer therapy in combination with strategies targeting tumor cells. For example, specifically targeting immune-suppressive CAFs may be combined with cell-mediated immuno-therapies. Therapeutic strategies using CAFs can be categorized into three groups: 1. Strategies directly targeting CAFs by eliminating them or preventing their activation; 2. Strategies targeting CAF activity by inhibiting factors they produce and their impact on cancer cells and TME cells [20,33,159]; and 3. Strategies taking advantage of the tumor-tropism of MSCs (CAF precursors) to deliver anti-neoplastic molecules to tumors. 

### 5.1. Strategies Directly Targeting CAFs

Strategies to eliminate CAFs have been limited by the need to identify a target molecule expressed on their surface and the lack of markers specific for CAFs as discussed earlier. Approaches targeting αFAP have been the most widely tested as this protein is expressed in 90% of CAFs [160]. In murine models of breast, colon and lung cancers, and in human xenograft models of lung, colon, PDAC and OSCC cancers, PT-630, a small molecule inhibiting the peptidyl peptidase activity of αFAP, and anti-αFAP monoclonal antibodies have been used. They induce apoptosis in CAFs, and subsequently promote Th1 lymphocyte polarization and cytotoxic T lymphocytes (CTL) infiltration in tumors, thereby decreasing metastasis and tumor growth [116,161,162,163,164]. Chimeric antigen receptor T-cells (CAR-T) engineered against αFAP have shown significant efficacy without any toxicity in murine models of lung, breast and colon cancers [165]. When tested in Phase I clinical trials in non-small cell lung carcinoma (NCT00243204, NCT02209727), melanoma (NCT00083252) and pancreatic carcinoma (NCT00116389), sibrotuzumab, a monoclonal antibody against αFAP, and PT-100, a small molecule peptidyl peptidase inhibitor, have shown feasibility in the absence of severe toxicity, but their efficacy in Phase II trials has been limited [166,167,168]. TRC-105, a monoclonal antibody against endoglin (CD105) present on the surface of ECs, CAFs and MSCs, enhances immunotherapy with NK cells in xenotransplanted human neuroblastoma tumors [123]. It has also been tested in multiple Phase I, II and III trials as an anti-angiogenic agent, often in combination with bevacizumab (anti-VEFG), but has not yet been tested specifically for its activity against CAFs [169]. 

Strategies to prevent or reverse CAFs activation have also been developed. PDGF and TGF-β are the two main growth factors that activate CAFs. Dasatinib, a broad receptor tyrosine kinase inhibitor with activity against PDGFRβ, induces a reversion from the activated phenotype of CAFs to a phenotype closer to that of normal fibroblasts, limiting their proliferation [170]. Evidence that CAF activation involves regulatory miRNAs such as miR-221-5p, miR-145 and miR-Let-7 and epigenetic mechanisms such as DNA methylation and histone acetylation [171,172] suggests other strategies to reverse CAF activation. One such strategy tested in breast, prostate and OSCC cancer xenograft models is to re-induce the expression of miRNAs (miR-145, miR-15, miR-16, Let-7b) that prevent CAF activation. Transfection of these miRs into CAFs inhibits TGF-β activation and their secretion of IL-8 and FGF, decreasing tumor growth in murine models [173,174,175]. Another strategy is to use a histone deacetylase (HDAC) inhibitor such as scriptaid, which acts as a repressor of TGF-β activity [176]. In a murine melanoma model, scriptaid inhibits the activation of α-SMA+ and COL1+ CAFs by repressing TGF-β signaling and inhibiting ECM production and tumor invasion in a 3D model of tumor/CAFs, and delaying tumor growth in vivo in murine model [177]. The drug was well-tolerated and could be used as an adjunct to improve chemotherapy [178]. LIF activates JAK/STAT-3 and upregulates DNA methyl transferase (DNMT), inducing epigenetic changes in CAFs that promote their activation and migration. Accordingly, the combination of a JAK inhibitor (ruxolitinib) and a DNMT inhibitor (5-azacytidine) has been shown to inhibit CAFs activation [179]. Ruxolitinib has been tested in multiple Phase I and II clinical trials in pancreatic and lung cancers in combination with other tyrosine kinase inhibitors such as capecitabine (pancreatic) and afatinib and erlotinib (lung) [180,181,182]. These trials show generally good tolerance of this combination, but the efficiency is mostly moderate. Ruxolitinib improves survival for patients with metastatic pancreatic cancer, whereas in lung cancer, combining ruxolitinib with other drugs does not improve efficiency over administering ruxolitinib alone (NCT01423604, NCT02145637, NCT02155465) [180,181,182].

Based on data demonstrating a difference in the dependence of CAFs on BCL-2 vs. MCL-1 for survival, studies have shown that CAFs in human breast cancer are sensitive to inhibitors targeting MCL-1 (A-1210477 and S63845) but not to those targeting BCL-2/BCL-XL (ABT-737 and navitoclax) [91]. However, illustrating the issue of heterogeneity, the opposite effect was reported in cholangiocarcinoma and ovarian cancer [183,184,185].

### 5.2. Inhibitors of CAF Activity 

Targeting proteins secreted by CAFs when activated or the signaling pathways activated by these proteins in cancer or host cells in the TME is another line of research. Multiple small molecule inhibitors or blocking monoclonal antibodies against ECM molecules, cytokines, chemokines, growth factors and signaling pathways have been developed over the last several decades and have been tested in clinical trials, several with major success, others with disappointing results, especially when used alone. Resistance developed towards these molecules and pathways has been a major problem. 

The stromal collagen barrier produced by CAFs may limit drug bioavailability. Inhibitors targeting different components of the ECM have been developed to decrease the impact of CAFs on chemoresistance. For example, the renin-angiotensin system affects the proliferation, metabolism and growth of tumors, and the activation of CAFs, inducing fibrosis. Angiotensin receptor blockers (ARB) such as losartan have been shown to improve the bioavailability of drugs such as doxorubicin and nanoparticles in xenotransplanted models of pancreatic cancer and melanoma by decreasing the percentage of α-SMA+ cells and the amount of collagen in the tumor stroma, and the tumor size in murine models [186,187]. Clinical trials with ARB in combination with bevacizumab or FOLFIRINOX in pancreatic and colorectal cancers have shown prolonged progression-free survival (PFS) and overall survival (OS) when used as adjuvant therapy [188,189]. For example, Phase II clinical trials (NCT01821729, NCT03563248) in pancreatic cancer using a combination of losartan and FOLFIRINOX recently showed extended patient prognosis and was associated with negative resection margin for 61% of patients [189]. The administration of nab-paclitaxel (abraxane) combined with gemcitabine decreases the production of type-I collagen and the secretion of CXCL10 and IL-6 in co-cultures of pancreatic cells and CAFs [190,191]. A recent Phase III clinical trial tested nab-paclitaxel in combination with atezolizumab, a monoclonal antibody against PD-L1, in unresectable and metastatic triple-negative breast cancer (NCT02425891) and metastatic lung cancer (NCT02367781), showing an increase in PFS and OS [192,193,194]. These inhibitors, which have an effect on ECM, could preferentially target CAF subtypes implicated in ECM production and remodeling such as CAF-N, CAF-A and myCAFs.

Multiple inhibitors of growth factors, cytokines and chemokines produced by CAFs in the TME have been tested in pre-clinical models and several have reached clinical trials [33,159]. For example, CXCL-12 and TGF-β are major factors secreted by CAFs that recruit immune cells and ECs into tumors and stimulate EMT and proliferation in cancer cells. Plerixafor (AMD3100), a small molecule inhibitor for CXCR4, the receptor for CXCL-12, has been tested in murine models of prostate cancers and PDAC. In the KPC murine model of PDAC, AMD3100 in combination with ipilimumab, a monoclonal antibody against the immune checkpoint, CTLA-4, inhibits αFAP+ CAFs and increases the recruitment of CTL [195,196,197]. Plerixafor is currently undergoing clinical trials for PDAC, colorectal and ovarian cancers (NCT03277209) [197]. Two clinical trials combining plerixafor with antibodies against PD-1, i.e., cemiplimab in metastatic PDAC (NCT04177810) and pembrolizumab in head and neck carcinoma (NCT04058145) are ongoing. Galunisertib (LY3200882), a small molecule targeting the TGF-β receptor has been the focus of multiple pre-clinical and clinical studies. In murine models of breast and liver cancers, treatment with galunisertib inhibits the growth of tumors by increasing the infiltration of CD8+ T lymphocytes, and their invasion by increasing the accumulation of collagen and by inhibiting EMT [198,199]. Treatment with galunisertib in combination with gemcitabine in a Phase Ib/II trial in patients with PDAC resulted in an increase in overall survival when compared to gemcitabine alone (NCT01373164) [200]. Other clinical trials testing galunisertib in combination with capecitabine, an anti-metabolite drug, in colorectal cancer (NCT04031872), or with enzalutamide, an androgen receptor inhibitor, in metastatic prostate cancer (NCT02452008) are ongoing. These inhibitors have the advantage of targeting both CAF activation and CAF activity. 

Thus, we can begin to envision how targeting CAFs may become more precise (Table 1). For example, an inhibitor of cytokine-mediated signaling, such as ruxolitinib, may be used to more specifically block the activity of iCAFs whereas an inhibitor of TGF-β, such as galunisertib, will preferentially target myCAFs and TGFβ-myCAFs.

### 5.3. Strategies Using CAF Precursors as Delivery Tools

A third strategy is to take advantage of the tumor-tropism of CAFs, and in particular MSCs, to deliver anti-tumor molecules. MSCs are attracted to the sites of tissue repair in diseases such as chronic inflammatory diseases, ischemic stroke and cancers. Three strategies are presently explored: (1) the use of MSCs grown in vitro as producers of a large amount of EVs that can be collected and used to deliver drugs to tumors in vivo [201]; (2) the use of MSCs amplified ex vivo and administered in vivo as vehicles of anti-tumor drugs; (3) the use of MSCs genetically modified ex vivo to produce molecules that reduce tumor progression and are administered in vivo [202,203]. 

MSCs-derived EVs (called iExosomes), carrying siRNA or shRNA against KRAS^G12D^, a mutation often found in cancers, have been tested in different murine models of PDAC, and shown to inhibit metastases and increase overall survival, particularly when administered in combination with an anti-CD47 (“don’t eat me” signal) monoclonal antibody that promotes RAS-dependent macropinocytosis of iExosomes. A clinical trial with these iExosomes is currently ongoing (NCT03608631) [204,205]. In melanoma and pancreatic cancers, MSCs delivering paclitaxel induce a decrease of the proliferation of tumor cells in vitro [206,207]. MSCs collected from patients and engineered ex-vivo can be safely and efficiently re-administered to the patient [208]. A clinical study in metastatic gastrointestinal cancer has shown that engineered MSCs are localized at the site of the primary cancer tumor [208]. In mouse prostate and metastasis lung models, MSCs engineered to overexpress IFNβ have an antitumorigenic effect by inducing cell death by apoptosis, stimulating the cytotoxic activity of NK cells, and inhibiting angiogenesis [209]. A clinical trial with IFNβ-MSCs is currently recruiting patients with ovarian cancer (NCT02530047).

## 6. Conclusions

Our understanding of the contribution of CAFs to cancer progression has dramatically changed since their initial description in the early 1990s. We now have a much better understanding of their heterogeneity and the complexity of their functions and phenotypes. As we have become more proficient at precisely identifying subgroups of CAFs with specific functions and localization in tumors, it may soon be possible to design “molecularly-informed clinical trials” that combine the targeting of tumor cells with the targeting of subpopulations of CAFs that specifically provide tumor cells with mechanisms for therapeutic escape.

## Figures and Tables

**Table 1:** Phenotypic and functional heterogeneity of Cancer-Associated Fibroblasts and therapeutic approaches.

Cancers	Subtypes	Clusters	IHC/Flow Cytometry	scRNA-seq	Functions	Ref.	Preclinical/Clinical Trials	
							Not Specific	Probably Specific
Breast/PDAC	CAF-1		FSP1, VEGF, TNC		Angiogenesis, Metastasis	[54,55,56,57]	αFAP therapy, CD105 mAb, Dasatinib, miRNAs therapy, Scriptaid, Ruxolitinib, Losartan, Nab-paclitaxel, AMD3100, Galunisertib	
	CAF-2		αSMA, NG2, PDGFRβ		Physical Barrier, Immunosuppression			Dasatinib
OSCC	CAF-N		HA, MMP		Invasion, Immunosuppression	[58,59]		Losartan, Nab-paclitaxel
	CAF-D		TGF-β		Migration			Galunisertib
Colorectal	CAF-A			MMP2, αFAP, COL1A2		[60]		αFAP therapy, Losartan, Nab-paclitaxel
	CAF-B			αSMA, PDGFA, TAGLN				
PDAC	rCAFs		PDPN, meflin		Anti-tumorigenic	[61,62]		
	myCAFs (pCAFs)		PDPN, αSMA	αSMA, TAGLN, TPM1, TPM2, POSTN	Proliferation, Migration, Invasion, Metastasis	[63,64,65,66]		Galunisertib, Losartan, Nab-paclitaxel
		LRRC15	PDPN, αSMA, LRRC15		Chemoresistance	[66]		
	iCAFs (pCAFs)		PDPN, IL-6, LIF, IL-11	IL-6, IL-8, CXCL1, CXCL12, CFD, LMN, DPT	Metastasis, Angiogenesis, Immunosuppression	[63,64,65,66]		Ruxolitinib
	apCAFs (pCAFs)		PDPN, COL1A2	H2-Aa, H2-Ab1, CD74	Immunosuppression	[64,67]		
Breast	CAF-S1	ecm-myCAF	CD29, αFAP, PDGFRβ, FSP1, αSMA, cav1	LRRC15, GBJ2	Proliferation, Migration, Invasion, Metastasis, Immunosuppression	[68,69,70,71]		αFAP therapy (CAF-S1), Dasatinib (CAF-S1), Galunisertib (myCAF), Ruxolitinib (iCAF)
		detox-iCAF		ADH1B, GPX3				
		IL-iCAF		RGMA, SCARA5				
		TGFβ-myCAF		CST1, TGFβ1				
		wound-myCAF		SEMA3C, SFRP4				
		IFNγ-iCAF		CCL19, CCL5				
		IFNαβ-myCAF		IFIT3, IRF				
		acto-myCAF		GGH, PLP2				
	CAF-S2							
	CAF-S3		CD29, FSP1, PDGFRβ					Dasatinib
	CAF-S4		CD29, FSP1, PDGFRβ, αSMA		Proliferation, Migration, Invasion, Metastasis			Dasatinib
	CD10/GPR77		CD10, GPR77		Proliferation, Migration, Chemoresistance	[72]		
	vCAFs			Cdh5, Pecam1, CD34, Notch3, Nr2f2, Epas1	Angiogenesis	[73]		
	dCAFs			MFAP5, Scgr1, Sox9, Sox10				
	mCAFs			Dcn, Lum, Fbln1, Smoc, Lox, Loxl1				

**Table 2:** Different mechanisms of pro- and anti-tumorigenic CAFs activities with the secretion associated.

Activity	Mechanisms	Proteins involved	Ref.
Proliferation, Survival	Stimulation of proliferation	TGF-β1, CXCL-12, FGF, POSTN, OPN, HGF, IL-6, IL-22	[83,84,85,86,87,88,89,90]
	Inhibition of apoptosis	Upregulation of BCL-2 and MCL1, downregulation of Bax	[83,84]
Chemoresistance	Inhibition of apoptosis	IL-6, IL-17A, PDGF, IGF, upregulation of MCL-1	[91,92,93,94,95]
	Stimulation of CSCs	C5a, IL-6	[72,94]
	Inhibition of bioavailability, vascular collapse	HA, collagen	[96,97,98,99]
	Ferroptosis, cell cycle inhibition	miR-522, CmiR-98-5p	[100,101]
Migration, Invasion, Metastasis	Stimulation of EMT	TGF-β, IL-32, PDGF, FGF, HGF, C3a	[102,103,104,105]
	Stimulation of cytoskeleton (motility)	TGF-β, upregulation of ARHGAP29	[103,106]
	ECM remodeling	MMP2, MMP3, MMP9	[59,104]
Angiogenesis	Recruitment/Proliferation of ECs and pericytes	VEGF, PDGF, CXCL-12, HGF, IL-6, IL-8	[107,108,109,110,111]
	Vascular mimicry	TGF-β, CXCL-12, MMP2	[112,113]
Immunomodulation	Recruitment/Proliferation of immune cells	IL-1β, CCL22, CXCL-12, CCL2, CXCL1, CXCL5, IL-8, PGE2	[80,114,115]
	Polarization of immune cells	IL-10, IL-12	[116,117,118,119,120]
	Immunotolerance (MDSC, Treg…)	CCL17, CCL22, CCL2, CXCL-12, IL-6, IL-17, IL-10, PD-1, CTLA4	[68,70,71,80,81,82]
	Inhibition of cytotoxic cells (lymphocyte, NK cells…)	TGF-β, CXCL1, IL-10, βig-h3, IL-6, IL-17	[68,70,121,122,123,124,125]
	Antigen presenting	MHC-II, CD74	[64,67,71]
Anti-tumorigenic	Inhibition of proliferation	IL-6, TNF-α, TGF-β	[126,127,128,129,130,131]
	Inhibition of CSCs stimulation	BMP4	[132]
	Inhibition of angiogenesis	Downregulation of HGF, FGF, VEGF, IL-8	[126,127,133]
	Inhibition of Treg cells	Downregulation of HGF, IL-6, FGF, CXCL-12	[126,127,133]
